# Characterization of Aberrations in DNA Damage Repair Pathways in Gastrointestinal Stromal Tumors: The Clinicopathologic Relevance of γH2AX and 53BP1 in Correlation with Heterozygous Deletions of *CHEK2*, *BRCA2*, and *RB1*

**DOI:** 10.3390/cancers14071787

**Published:** 2022-03-31

**Authors:** Ting-Ting Liu, Chien-Feng Li, Kien-Thiam Tan, Yi-Hua Jan, Pei-Hang Lee, Chih-Hao Huang, Shih-Chen Yu, Cheng-Feng Tsao, Jui-Chu Wang, Hsuan-Ying Huang

**Affiliations:** 1 Department of Pathology, Kaohsiung Chang Gung Memorial Hospital and Chang Gung University College of Medicine, Kaohsiung 833, Taiwan; liutt107@cgmh.org.tw (T.-T.L.); mr9244@cgmh.org.tw (P.-H.L.); wingthink@cgmh.org.tw (C.-H.H.); yu5250@cgmh.org.tw (S.-C.Y.); juichu0918@cgmh.org.tw (J.-C.W.); 2 Department of Medical Laboratory Science, I-Shou University, Kaohsiung 833, Taiwan; 3 Department of Pathology, Chi-Mei Medical Center, Tainan 710, Taiwan; cfli@mail.chimei.org.tw; 4 National Institute of Cancer Research, National Health Research Institutes, Tainan 704, Taiwan; 5 Department of Biotechnology, Southern Taiwan University of Science and Technology, Tainan 710, Taiwan; 6 Department of Medical Informatic, ACT Genomics Co., Ltd., Taipei 100, Taiwan; jtchen@actgenomics.com (K.-T.T.); isaacjan@actgenomics.com (Y.-H.J.); 7 Department of Internal Medicine, Kaohsiung Chang Gung Memorial Hospital and Chang Gung University College of Medicine, Kaohsiung 833, Taiwan; a9650@cgmh.org.tw

**Keywords:** gastrointestinal stromal tumor, DNA damage repair, γ-H2AX, 53BP1, *BRCA2*, *CHEK2*, *RB1*, disease-free survival

## Abstract

**Simple Summary:**

Aberrations of DNA damage repair (DDR) pathways enable the transformation of pre-neoplastic lesions, but their roles are barely understood in gastrointestinal stromal tumors (GISTs). The targeted next-generation sequencing of GISTs has demonstrated heterozygous deletions (HetDels) as frequent aberrations of DDR genes, usually lacking recurrent pathogenic single-nucleotide variants. As independently validated by multiplex ligation-dependent probe amplification, *CHEK2*-HetDel was the most prevalent, and similar to *BRCA2*-HetDel, was only related to older age groups. *RB1*-HetDel, albeit rare, was preferentially detected in the non-gastric, high-risk, and 53BP1-overexpressing GISTs, with occasional co-occurrence with *BRCA2*-HetDel. Being strongly correlated with immunofluorescence, immunohistochemistry reliably showed positive associations between the risk levels and expression levels of γH2AX and 53BP1, representative of DDR response, in two independent cohorts. Compared with those overexpressing either one or two biomarkers, low expressers of both γH2AX and 53BP1 displayed significantly longer disease-free survival in GISTs, indicating the early engagement of DDR aberrations in tumorigenesis.

**Abstract:**

Genetic aberrations involving DNA damage repair (DDR) remain underexplored in gastrointestinal stromal tumors (GISTs). We characterized DDR abnormalities using targeted next-generation sequencing and multiplex ligation-dependent probe amplification, and performed immunofluorescence (IF) and immunohistochemistry (IHC) analyses of γH2AX and 53BP1. Consistent with IF-validated nuclear co-localization, γH2AX and 53BP1 showed robust correlations in expression levels, as did both biomarkers between IF and IHC. Without recurrent pathogenic single-nucleotide variants, heterozygous deletions (HetDels) frequently targeted DNA damage-sensing genes, with *CHEK2*-HetDel being the most prevalent. Despite their chromosomal proximity, *BRCA2* and *RB1* were occasionally hit by HetDels and were seldom co-deleted. HetDels of *CHEK2* and *BRCA2* showed a preference for older age groups, while *RB1*-HetDel predominated in the non-gastric, high-risk, and 53BP1-overexpressing GISTs. Higher risk levels were consistently related to γ-H2AX or 53BP1 overexpression (all *p* < 0.01) in two validation cohorts, while only 53BP1 overexpression was associated with the deletion of *KIT* exon 11 (*KIT*ex11-del) among genotyped GISTs. Low expressers of dual biomarkers were shown by univariate analysis to have longer disease-free survival (*p* = 0.031). However, higher risk levels, epithelioid histology, and *KIT*ex11-del retained prognostic independence. Conclusively, IHC is a useful surrogate of laborious IF in the combined assessment of 53BP1 and γ-H2AX to identify potential DDR-defective GISTs, which were frequently aberrated by HetDels and a harbinger of progression.

## 1. Introduction

Thought to differentiate into Cajal cells, the majority of gastrointestinal stromal tumors (GISTs) harbor mutually exclusive *KIT* or *PDGFRA* mutations, which drive tumorigenesis and dictate responses to imatinib [1,2,3]. Prognostically, the National Institute of Health (NIH) scheme and the National Comprehensive Cancer Network (NCCN) guidelines are deemed to be effective in risk stratification for *KIT/PDGFRA*-mutated GISTs, although the former overrates the aggressiveness of large, mitotically inactive GISTs of the stomach, and the latter lacks sufficient evidence-based data in some uncommon settings [1,3]. Among the remaining 10–15% of GISTs with wild-type *KIT* and *PDGFRA*, the majority of cases are deficient in the enzymes of the succinate dehydrogenase (SDH) complex, occur exclusively in the stomach, and have genetic associations with Carney triad or Carney–Stratakis syndrome [4,5], while the NIH and NCC criteria fail to provide effective prognostication [4,5,6].

More rarely, quadruple “wild-type” GISTs may harbor mutated *NF1*, *BRAF*, or *RAS* genes, or may carry fusion genes involving *NTRK3*, *FGFR1* or *BRAF* as the driver aberrations, while very little is known about their pathological features and clinical behavior [7,8,9]. To refine prognostication beyond histological assessment, it is desirable to identify the aberrations of non-kinase pathways that link to GIST inception and progression.

DNA damage, especially the most deleterious double-strand breaks (DSBs), poses a significant threat to the viability of normal cells, and leads to the loss of genetic material if replication is not halted in the presence of damaged DNA [10,11,12,13]. To effectively repair this damage, evolutionary adaptions have developed competing but interwoven repair pathways. Whether endogenous or exogenous in etiology, various mutations or copy number variants (CNVs) may aberrantly affect the expression of DNA damage repair (DDR)-regulating genes in tumor cells, and instigate genome instability (GIN) under selective pressure, a cancer hallmark leading to malignant transformation and evolution [12,13,14,15]. Upon DNA damage, DNA repair pathways are activated with phosphorylation at serine 139 of H2AX, forming the γ-H2AX foci [11,13]. At the sites of DNA breaks, γ-H2AX foci recruit P53-binding protein 1 (53BP1), BRCA1, MDC1, and the MRE11–RAD50–NBS1 complex, in which the NBS1, together with MER11, further activates ATM kinase to trigger the sequential engagement of various homologous recombination repair (HRR) proteins [13]. 53BP1 is an evolutionarily conserved DDR protein, which partakes in both HR and the error-prone NHEJ repair pathway and co-localizes with phosphorylated ATM at sites of DSBs to cooperatively activate P53, which in turn orchestrates various cellular processes [11,12,13].

Similar to γ-H2AX, 53BP1 may serve as a surrogate biomarker of DDR, given that endogenous large foci of γ-H2AX and 53BP1 are significantly linked to the evolution from precancerous to invasive lesions and/or poor prognosis in carcinogenesis [16,17,18]. However, it remains largely unclear whether DDR alterations, especially the expression status of γ-H2AX and 53BP1, play a role in the disease inception and progression of GISTs. In the non-hereditary neoplasia, HR genes, including *BRCA1*, *BRCA2,* and those beyond, hit by heterozygous deletion (HetDel) are not uncommonly detected by next-generation sequencing (NGS) assays, while their functional and clinical relevance remains underexplored [11,19,20]. Attributable to their close chromosomal proximity, one copy each of *BRCA2* on 13q13.1 and *RB1* on 13q14.2 can be concomitantly lost in prostate cancers [21]. Aside from 13q, 22q is also recurrently lost during the evolution of GISTs [22,23]. Notably, *CHEK2*, located on 22q12.1, is a crucial HR regulator found downstream of ATM kinase, but it remains insufficiently characterized to assert its clinical relevance in GISTs [24]. To interrogate the implications of the DDR gene aberrations underlying GIST pathogenesis and progression, we performed targeted next-generation sequencing (NGS), analyzed the expression status and clinicopathologic associations of γ-H2AX and 53BP1 using whole-tissue blocks and tissue microarrays (TMAs), and employed multiplex ligation-dependent probe amplification (MLPA) assays to profile CNVs of *BRCA2*, *CHEK2*, and *RB1* in GISTs, to derive correlation with immunohistochemistry (IHC) and immunofluorescence (IF) results for γ-H2AX and 53BP1. The prognostic utility of the immunohistochemical expression of γ-H2AX and 53BP1 in predicting disease-free survival (DFS) was independently examined in a large TMA-based cohort of molecularly characterized GISTs. Our findings shed light on the potential engagement of DDR genetic aberrations and the clinicopathologic relevance of the heightened expression of γ-H2AX and 53BP1 in GISTs, which represent useful biomarkers of aggressiveness in tumor evolution and aid in distinguishing cases at risk of progression.

## 2. Materials and Methods

### 2.1. Study Cohorts

**Figure 1:**
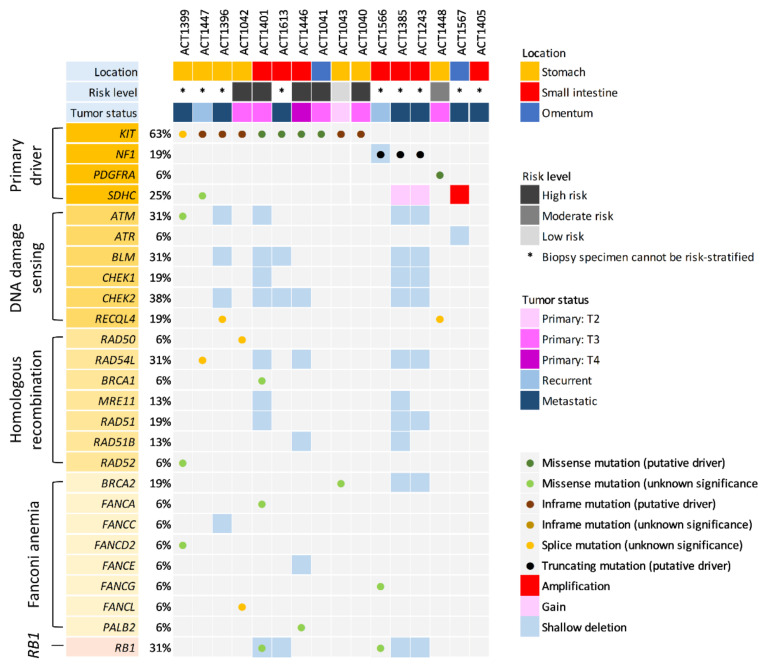
Mutational landscape of GISTs in *RB1* and homologous recombination repair-related genes: Types of aberrations are annotated and catalogued into DNA damage-sensing, homologous recombination, and Fanconi anemia pathways, and *RB1* as indicated by the color codes at the bottom. Note that DNA damage-sensing genes, e.g., *CHEK2*, are preferentially targeted by heterozygous deletion (HetDel) in contrast to another two groups mainly affected by isolated SNVs, such as missense or splicing mutations. Note that 2 of 16 samples exhibited co-occurring HetDels of *RB1* and *BRCA2* with close proximity to each other on 13q. Columns represent individual tumors and rows represent individual aberrated genes. DNA damage repair genes are collated based on the prevalence of CNVs and SNVs/indels, following the primary GIST-driving mutations (*KIT*, *PDGFRA*, *NF1*, *SDHC*) and the baseline clinicopathologic characteristics, including tumor locations, NCCN risk levels, and status of tumor specimens. Asterisks indicate biopsy samples not amenable to precise risk-stratification by histology.

This study (201901147B0) was approved by the institutional review board of Chang Gung Hospital. Targeted NGS (Figure 1) was performed to profile genetic aberrations in 16 GISTs, consisting of 7 primary resected samples (4 gastric, 2 intestinal, 1 omental) and 9 biopsy specimens, including 2 recurrent lesions (1 gastric, 1 intestinal) and 7 metastatic/disseminated GISTs. Of the primary resected samples, there were 1, 1, and 5 cases classified low-risk, moderate-risk, and high-risk, respectively. Although risk-stratification could not be effectively determined, the biopsy specimens could be considered together with high-risk GISTs, given their recurrent or metastatic nature. For IHC and IF analyses, the expression levels of γ-H2AX and 53BP1 were assessed on sections recut from a representative paraffin-embedded block of each case in another 84 resected GISTs as the training cohort, 41 of which were subjected to MLPA assays in parallel. In an independent validation cohort of 285 primary GISTs resected before 2013, tissue microarrays (TMA) were reconstructed to assemble tissue cores (1.5 mm) in triplicate for each sample. Having been previously characterized for mutations in *KIT*, *PDGFRA*, or v-raf murine sarcoma viral oncogene homolog B (*BRAF*) [25], the TMA-based cases were recut to perform the immunohistochemical staining of γ-H2AX and 53BP1 to determine clinicopathologic and prognostic correlations.

### 2.2. Targeted NGS

Paraffin-embedded blocks of tumor tissues, along with adjacent non-tumoral tissues when available, were recut to obtain ten 10 μm-thick sections for genomic DNA extraction using the RecoverAll Total Nucleic Acid Isolation Kit (Thermo Fisher, Waltham, MA, USA). The quality and yield of the extracted DNA were determined by Qubit™ dsDNA HS and Nanodrop analyses, respectively. The barcoded libraries were enriched by emulsion PCR following the manufacturer’s instructions. DNA amplification and sequencing were performed using the Ion AmpliSeq Comprehensive Cancer Panel of 440 cancer-related genes and an Ion Proton sequencer with an Ion P1 chip (Life Technologies, Carlsbad, CA, USA), respectively. The mean sequencing depth was set at >500×. For variant analysis, the human genome sequence hg19, the Torrent Suite Server version 5.0 and the Torrent Suite Variant Caller plug-in version 5.0 were used.

We screened the collection of base substitutions based on predicted impact on gene function, as well as known allelic frequencies in control populations, to identify possible germline and single-nucleotide polymorphisms in the PopFeqMax database from the ANNOVAR package, which integrates allelic frequencies (AF) from the 1000 Genomes Project. The set of mutations was further filtered for known roles in human cancers. Variants with a frequency of ≥5% were adopted for further analysis. SNVs, small indels, and CNVs were analyzed by ONCOCNV (https://github.com/BoevaLab/ONCOCNV, accessed on 18 February 2022). Mutations were construed as deleterious if they were cancer-recurrent variants with corresponding COSMIC IDs, frameshift, nonsense and splice-site mutations, or as probably damaging depending on the annotation of OncoKB knowledge database as well as the prediction in silico by the SIFT (https://sift.bii.a-star.edu.sg/, accessed on 18 February 2022), PolyPhen2 (http://genetics.bwh.harvard.edu/pph2/, accessed on 18 February 2022), or Grantham (https://ionreporter.thermofisher.com/ionreporter/help/GUID-D9DFB21C-652D-4F95-8132-0C442F65399.html, accessed on 18 February 2022) platforms. However, variants construed as possibly damaging were classified as variants of unknown significance. Thorough searches of the literature in the COSMIC and ClinVar databases were performed to enumerate significantly mutated genes, focusing on those involved in DDR pathways.

### 2.3. IHC Analysis for γH2AX and 53BP1 in GISTs

Whole tissue sections were recut from 84 GISTs of the training cohort and 20 samples of adjacent gastrointestinal muscular tissue, pressure-cooked in 10 mM citrate buffer (pH6) for 7 min for antigen retrieval, washed with TBS buffer, treated with 3% H_2_O_2_ to quench endogenous peroxidase, and incubated with primary antibodies against γH2AX (mouse monoclonal, 1:100, Abcam, Cambridge, UK) and 53BP1 (rabbit monoclonal, 1:500, Abcam, Cambridge, UK). The ChemMate DAKO EnVision kit was used to detect reactivity to 53BP1 and γH2AX in the tumoral nuclei of GISTs. Two pathologists (T.T.L, L.C.F) blinded to the data of the clinicopathologic variables independently assessed the nuclear expression of γH2AX and 53BP1 using the previously applied H-score method [25], which is defined by the equation, ΣP_i_ (i + 1) where i is the intensity of stained tumor cells (0–3+) and P_i_ is the percentage of stained tumor cells ranging from 0% to 100%. Contradictory cases were reviewed by the senior author to obtain consensus. Using their median values as the cutoffs, 84 GISTs were dichotomized into high and low expressers of γ-H2AX and 53BP1, according to the H-scores, by IHC.

### 2.4. IF Analyses of Expression Levels and Subcellular Spatial Association of γH2AX and 53BP1 in GISTs

IF staining was performed on the same set of GISTs and normal tissues as was used in the whole sections stained with IHC. Briefly, deparaffinization, microwave-heated antigen retrieval in DAKO buffer, immersion with 10% normal goat serum, and incubation with anti-γH2AX (rabbit monoclonal, 1:500, Abcam, Cambridge, UK) or anti-53BP1 (mouse monoclonal, 1:500, Abcam, Cambridge, UK) for one hour at room temperature, were processed using standard procedures. The slides were then incubated with Alexa Fluor 568-conjugated goat anti-rabbit antibody (Perkin Elmer, Boston, MA, USA) for both γH2AX and 53BP1 to assess their expression levels. In 8 cases selected for dual IF assays, additional slides were incubated with goat anti-rabbit antibodies conjugated with Alexa Fluor 568 and Alexa Fluor 488 for γ-H2AX and 53BP1, respectively, to visualize their nuclear co-localization. Slides were DAPI-counterstained and photographed using a Nikon microscope (Nikon, Tokyo, Japan) under a 100 X-immersion oil lens in the Z-stack mode. The γH2AX or 53BP1 immunoreactivity pattern was considered positive when tumor cells exhibited concrete foci >1.0 μm in their short axes [17], while faint or hazy nuclear staining was disregarded. The expression percentages of GIST tumor cells and normal smooth muscle cells exhibiting foci of γH2AX or 53BP1 staining patterns were recorded for a minimum of 100 cells in each sample. Using their median values as the cutoffs, 84 GISTs were dichotomized into high and low expressers of γ-H2AX and 53BP1, based on percentages of cells with reactive foci, by IF.

### 2.5. MLPA

Following the manufacturer’s instructions (MRC-Holland), MLPA assays targeting *BRCA2*/*CHEK2* (SALA MLPA Probemix P045-D1) and *RB1* (SALA MLPA Probemix P047) were performed in 41 samples of the training cohort and 3 normal gastrointestinal muscular tissues as the reference.

Briefly, 100–200 ng of genomic DNA in the TE Buffer was denatured for 5 min at 98 °C, followed by the addition of SALSA MLPA buffer and the MLPA probes and incubation for 1 min at 95 °C, allowing the probes to hybridize to their respective targets for 16 h at 60 °C. Ligation of the hybridized probes was carried out for 15 min by lowering the temperature to 54 °C and adding Ligase-65 mix. The ligase mix, after inactivating the enzyme at 98 °C, was diluted with PCR buffer and then supplemented with Universal PCR primers and SALSA polymerase. In each ligation reaction, VAPOR-LOCK was employed to prevent evaporation effects with all ligations being performed in duplicate. The PCR reaction was performed using the ABI9700 thermal cycler. The PCR amplification of the ligated MLPA probes followed the scheme of 35 cycles of 30 s at 95 °C, 30 s at 60 °C and 1 min at 72 °C, and the PCR fragments were purified, supplemented with LIZ500 size standard, and analyzed with capillary gel electrophoresis by an ABI3730 DNA analyzer (Applied Biosystems, Foster City, CA, USA) according to the length of products.

In each sample, the peak areas achieved with specific probes for individual exons in genes of interest were first normalized by the averages of peak areas derived by probes targeting loci from different chromosomes that had been properly modified as references based on their amplicon sizes. A corresponding calculation was performed on the pooled reference DNA from adjacent non-neoplastic tissue samples. A corresponding calculation was derived from the pooled reference DNA from adjacent non-neoplastic tissue samples. A final ratio was then determined by dividing the value of the tumor samples by the value of the pooled reference control [26]. Regarding the *RB1* and *BRCA2* genes, the median values across all specific probes were calculated for each sample. If the median dosage quotient (DQ) was equal to or near zero, the sample was construed as exhibiting homozygous deletion, while median DQs of ≤0.7 and ≥1.3 were interpreted as HetDel and heterozygous duplication, respectively [26]. For the *CHEK2* gene, any probe with DQ ≤0.7 or ≥1.3 was considered heterozygously deleted or heterozygously duplicated, respectively, as only probes for the representative exons 1 and 9 are included in the MLPA kit.

### 2.6. Statistical Analysis

In the training cohort, 84 GISTs were used in evaluating the associations between IHC H-scores and the IF expression percentages of γ-H2AX or 53BP1 by Pearson correlative analyses, and those between γ-H2AX and 53BP1 assessed by either IHC or IF. A Mann–Whitney U test was applied to compare the differences in the IHC H-scores and the IF expression percentages of γH2AX and 53BP1 between the normal and GIST tissues; among individual risk levels defined by NIH scheme or NCCN guidelines; and between the joint no-/very low-/low-risk and moderate-/high-risk groups. The findings of γ-H2AX and 53BP1 by IHC or IF in both the training and TMA-based validation cohorts were further correlated with clinicopathologic variables, including NIH- and NCCN-defined risk levels, using the Chi-square/Fisher exact and Wilcoxon rank-sum tests for categorical and continuous variables, respectively. Follow-up data regarding the survival period were available for 285 primary imatinib-naïve GISTs in the TMA cohort (median period: 61.9 months; range: 1–234 months). The endpoint was disease-free survival (DFS) that was unaffected by adjuvant imatinib therapy for relapsed tumors. To facilitate the prognostic correlations with DFS, 285 GISTs were categorized as those with the 5′ deletion of the *KIT* exon 11 (*n* = 83) and those without (*n* = 202).

## 3. Results

### 3.1. Targeted NGS for Primary Drivers and a Focused Appraisal of DDR Genes

In 16 cases profiled by targeted NGS (Figure 1), 10 GISTs harbored various *KIT* mutations, including primary pathogenic drivers in nine cases and a splicing mutation of unknown significance in one. Corresponding to the latter exceptional case, *KIT* deletions at intron 10−exon 11 have been reported in 2.6% of all GISTs that lost the normal splice acceptor site at the beginning of exon 11 [27]. Other driver mutations included *PDGRA* mutation (p.D842Y) in one gastric GIST, and *NF1* mutations (p.Q1815 *: *n* = 2, p.F1247fs: *n* = 1) in three small intestinal cases. Another two cases were wild-type for *KIT/PDGFRA/BRAF/NF1*. Notably, a primary gastric GIST with in-frame *KIT* p.V560del also harbored a missense *SDHC* p.T32N SNV of unknown pathogenic significance, while the amplification (CNV = 7) and gain (CNV = 4) of *SDHC* were detected in one wild-type GIST and two GISTs with truncated NF1, respectively. Among the DDR genes, the DNA damage-sensing *CHEK2* was the most frequently altered, exhibiting HetDel in six cases (37.8%), while homozygous deletion, SNV or small INDEL of *CHEK2* was not detected in any case. *BRCA2* was hit by HetDel in two cases and by missense mutation (p.G2508S) in another. *RB1*-HetDel was detected in four cases (25%), among which *BRCA2*-HetDel co-occurred in two cases and *RB1* missense SNV (p.E492K) in another. In a single case, a standalone *RB1* missense SNV (p.R320G), without co-occurring *RB1*-HetDel, was identified and interpreted as a damaging/deleterious alteration by both Grantham and SIFT platforms.

In addition, HetDels of DDR genes also occurred in *BLM* (BLM RecQ like helicase, *n* = 5), *ATM* (ataxia-telangiectasia-mutated, *n* = 4), *CHEK1* (checkpoint kinase 1, *n* = 3), and *ATR* (ataxia-telangiectasia and Rad3-related, *n* = 1) in the damage-sensing pathway. Regarding the HRR pathway, *RAD51* was affected by HetDel in three cases, wherein one and two cases displayed concomitant *RAD51B*-HetDel and *MER11*-HetDel, respectively. However, these genes with recurrent HetDels very rarely exhibited SNVs (e.g., *ATM* in four cases with standalone HetDel vs. a single case with a standalone I2185T missense SNV), regardless of whether they occurred separately across different cases or concomitantly in identical cases.

Except for the likely benign c.1621-4G>C alteration at the splicing site of *RECQL4*, which occurred in two cases, other SNVs in genes involving DNA damage-sensing and HRR pathways were all detected in single samples. These included, each in one case, mid-exonic missense SNVs of *BRCA1* (p.G1350C), *RAD52* (p.V374I), *PALB2* (p.R825T), *FANCA* (p.R1409W), *FANCD2* (p.A439V), and *FANCG* (p.S603F), as well as splicing SNVs of *RAD50* (c.-204-5C>T), *RAD54L* (c.1170-8T>C), and *FANCL* (c.155+7T>C). However, the pathogenicity of these aberrations was classified as either holding unknown significance or being likely benign by Clinvar.

Aberrations of nucleotide excision repair (NER) and non-homologous end-joining (NHEJ) repair-associated genes were recurrently identified in four and three cases, respectively (Figure S1), and all were targeted by various missense SNVs, including *ERCC3* p.K11E (*n* = 2) and p.S764L (*n* = 1, pathogenic by COSMIC_ID3042186) and *ERCC5* p.R71H (*n* = 1) in the NER pathway and *PRKDC* p.M3846I, p.P2456A, and p.G1030V (*n* = 1 each) in the NHEJ pathway.

### 3.2. Expression Status of γ-H2AX and 53BP1 by IHC and IF in the Training Cohort

**Figure 2:**
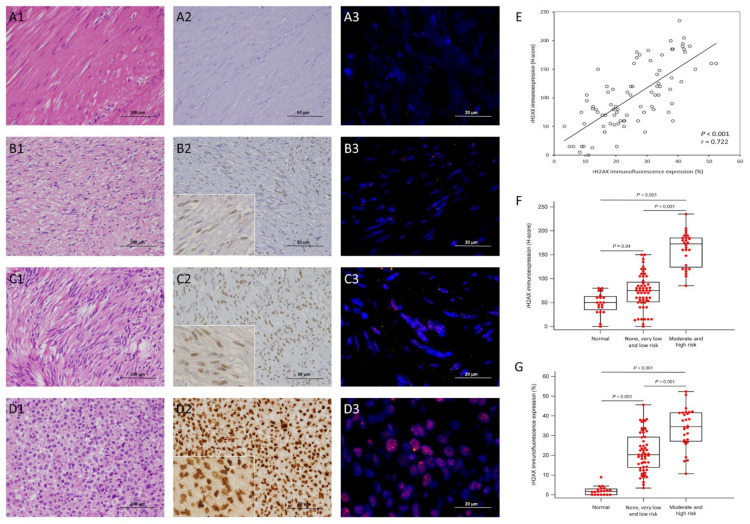
Immunohistochemical and immunofluorescence expressions of rH2AX in gastrointestinal stromal tumors across various risk categories. Representative adjacent normal tissue (**A1**) and gastrointestinal stromal tumors (GISTs) at low-risk (**B1**), moderate-risk (**C1**), and high-risk (**D1**) levels exhibited no (**A2**) to weak (**B2**), moderate (**C2**), and diffuse strong (**D2**) immunohistochemical (IHC) nuclear rH2AX reactivity (×200), respectively, in a granular pattern, as highlighted in the insets (**B2**–**D2**, ×400). In the immunofluorescence analysis of rH2AX, the percentages of positively labeled nuclei, presenting as fine to coarse red speckles, increased gradually from normal (**A3**) to low-risk (**B3**), moderate-risk (**C3**) and high-risk (**D3**) GISTs. (**E**) The scatter plot reliably demonstrates a strong association between the H-score of rH2AX immunoexpression on the *Y*-axis and immunofluorescence expression on the *X*-axis (*p* < 0.001, r = 0.722). Compared to the normal tissues, the expression levels of rH2AX determined by IHC ((**F**)*, p* < 0.001) and IF ((**G**), *p* < 0.001) assays were significantly elevated in the 84 GISTs; the major differences occurred in normal tissues versus none/very low-/low-risk cases (IHC, *p* = 0.04; IF, *p* < 0.001), and in the latter merged group versus the combined moderate/high-risk GISTs (*p* < 0.001 for both IHC and IF).

**Figure 3:**
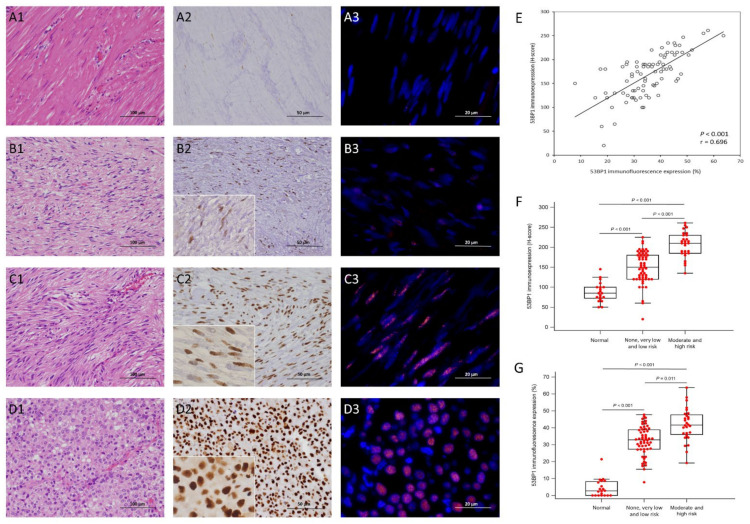
Immunohistochemical and immunofluorescence expression of 53BP1 in GISTs across various risk categories. Compared with the adjacent normal tissue (**A1**) without very limited and weak nuclear reactivity (**A2**), representative GISTs showed gradually increasing cellularity and greater epithelioid cytomorphology from the low-risk (**B1**) to the moderate-risk (**C1**) and high-risk (**D1**) cases, via histology (×200), and also exhibited focal (**B2**), moderate (**C2**) and diffuse strong (**D2**) nuclear 53BP1 expression, via immunohistochemistry (IHC), in a granular pattern (insets of **B2**–**D2**, ×400). Compared with the scarce and trivial dot-like signals indicative of very low endogenous expressions in adjacent normal tissue (**A3**), the immunofluorescence (IF) analysis of 53BP1, similar to IHC, revealed gradually increasing percentages of positively labeled nuclei from the low-risk (**B3**) to the moderate-risk (**C3**) and high-risk (**D3**) GISTs, respectively. (**E**) The scatter plot reliably exhibits a strong correlation between the H-score of 53BP1 immunoexpression on the *Y*-axis and immunofluorescence expression on the *X*-axis (*p* < 0.001, r = 0.696). The bar charts show similarly and overtly elevated expressions of nuclear 53BP1 in the H-score via IHC ((**F**), *p* < 0.001), and in the percentages of positive cells using IF ((**G**), *p* < 0.001), resulting from the increased levels of the 84 GISTs, wherein the differences were all strongly significant between normal tissue and none/very low-/low-risk cases, and between the latter merged group and the joint group of moderate/high-risk GISTs (*p* < 0.001 in all subgroup comparisons via IHC and IF).

In all 84 informative GISTs of the training cohort, there were 49 males and 35 females aged between 30 and 86 years (mean: 62; median: 64), whose tumors were located in the stomach and intestine in 58 and 26 cases, respectively, and were classified by the NIH scheme as high risk, intermediate risk, and low/very low risk, in 19, 17, and 48 cases, and by NCCN guidelines as high, moderate, low, and none/very low risk in 17, 11, 16, and 40 cases, respectively (Figure 2A1–D1 and Figure 3A1–D1).

Relative to normal tissues, the GISTs across all the risk levels defined by NCCN guidelines exhibited significantly higher immunohistochemical H-scores of γ-H2AX (*p*< 0.001, Figure 2A2–D2,F), but the γ-H2AX H-scores did not significantly vary between the high-risk and moderate-risk cases, nor did they exhibit significant differences among the cases classified as none, very low risk, and low risk. In this context, none, very low-, and low-risk cases were combined into a joint group, as were the moderate- and high-risk cases. The γ-H2AX H-score of the combined none/very low-/low-risk cases was not only significantly higher than that of adjacent normal tissues (*p* = 0.04), but was also noticeably lower than that of the joint group of GISTs at the high and moderate risk levels (*p* < 0.001). As shown in Table 1, high γ-H2AX expressers assessed by IHC were found to be significantly more common in intestinal (*p* = 0.005), mitotically active (*p* < 0.001), and larger GISTs (as a continuous parameter: *p* = 0.01; dichotomized at 5 cm: *p* = 0.018). The IF staining of γ-H2AX (Figure 2A3–D3,G) showed only scarce foci of γ-H2AX in a very low percentage (generally <3%, Figure 2A3) of smooth muscle cells in the adjacent normal tissues, with one outlier at 10%. In contrast, the expression percentages of γ-H2AX foci in tumor cells of GISTs were not only significantly higher in all NCCN-defined risk levels than those in normal tissue (*p* < 0.001), but they also exhibited significant stepwise increments from normal tissue to the joint none/very low-/low-risk group (*p* < 0.001), and from the latter to the joint group of moderate-risk and high-risk cases (*p* < 0.001), although there were no significant differences when individually comparing the cases at different risk levels within either of the two joint groups (Figure 2G). Notably, the γ-H2AX H-score assessed by IHC, and the expression percentage of γ-H2AX foci assessed by IF, exhibited a robustly positive correlation (*p* < 0.001, r = 0.722, Figure 2E), indicating the utility of IHC as a potential surrogate of IF for γ-H2AX. Although the high γ-H2AX expressers assessed by IF were not related to tumor location or size, these tumors exhibited significantly higher mitotic activity (*p* < 0.001). According to both the NIH scheme and the NCCN guidelines, significantly increased proportions of GISTs with elevated risk levels were found when using either IHC or IF (all *p* ≤ 0.001, Table 1).

Compared with the adjacent normal tissues, GISTs at all NIH-defined risk levels exhibited noticeably higher immunohistochemical 53BP1 H-scores (*p* < 0.001, Figure 3A2–D2,F). The differences were also equally robust in the comparisons between normal tissue and the joint group of none/very low-/low-risk cases (*p* < 0.001), and between two joint groups, namely, the none/very low-/low-risk versus moderate/high-risk cases (*p* < 0.001). As shown in Table 1, high 53BP1 expressers assessed by IHC exhibited significantly higher mitotic activity (*p* = 0.016) and increased proportions of cases classified into higher risk levels by both the NIH risk scheme and the NCCN guidelines (both *p* ≤ 0.001), but they showed no association with tumor size or location. Regarding the IF staining of 53BP1 (Figure 3A3–D3,G), only a few foci of 53BP1 were identified in a low percentage (generally <10%, Figure 3A3) of the adjacent smooth muscle cells, except for one outlier reaching 20%. Compared with the adjacent smooth muscle samples, the expression percentages of 53BP1 foci (Figure 3G) in GISTs were significantly higher across all NCCN-defined risk levels (*p* < 0.001) and in the joint group of none/very low-/low-risk cases (*p* < 0.001). Despite being slightly weaker in statistical power (*p* = 0.011), the joint group of moderate- and high-risk cases also showed significantly increased expression percentages of 53BP1 by IF than the joint group of none/very low/low-risk cases. Similar to 53BP1 IHC, high 53BP1 expressers assessed by IF also exhibited significantly higher mitotic activity (*p* < 0.001), and increased proportions of cases classified into higher risk levels by both the NIH risk scheme (*p* = 0.007) and the NCCN guidelines (*p* = 0.002). Notably, the IHC H-score of 53BP1 and the IF expression percentage of the 53BP1 foci exhibited a strongly positive correlation (*p* < 0.001, r = 0.696, Figure 3E).

Consistent with the role of 53BP1, in cooperating with γ-H2AX, in signifying DNA repair and GIN, the above findings imply that increased expressions of γ-H2AX and 53BP1 constitute an early event in the evolution of GISTs. In essence, γ-H2AX and 53BP1 began accumulating in the very low/low-risk cases and began increasing to relatively steady expression levels in GISTs in the moderate-risk group, which did not differ significantly from the high-risk ones.

### 3.3. Correlation and Co-Localization of γ-H2AX and 53BP1

**Figure 4:**
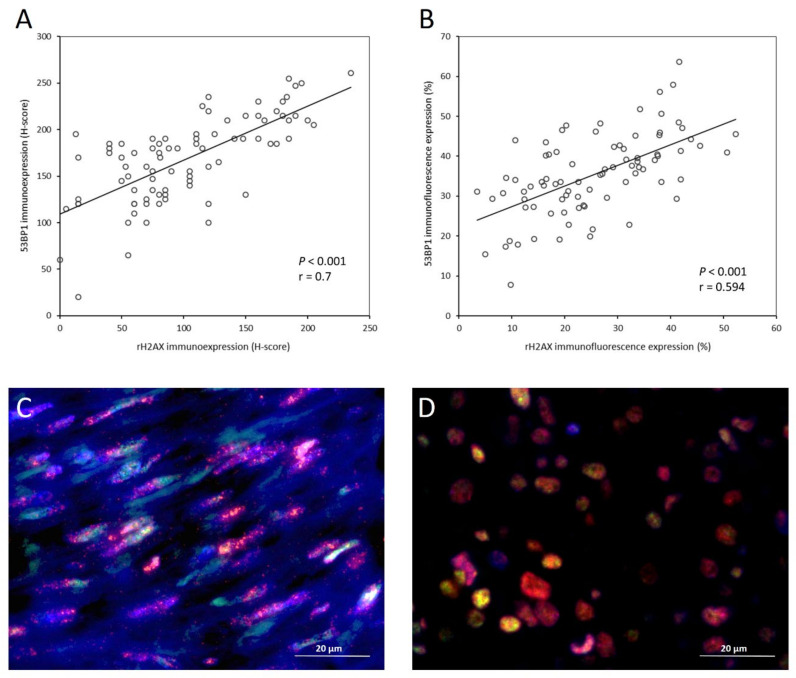
Strong associations between rH2AX and 53BP1 in expression levels and subcellular localization. The scatter plots of the H-scores of immunoexpression ((**A**), *p* < 0.001, r = 0.700) and the percentages of immunofluorescence expression ((**B**), *p* < 0.001, r = 0.594) both demonstrate strong correlations between rH2AX and 53BP1. Double immunofluorescence stains for rH2AX (red) and 53BP1 (green) with DAPI counterstaining (blue) in representative moderate-risk (**C**) and high-risk (**D**) GISTs showed granular yellow foci, indicative of colocalization, in the tumoral nuclei of the merged images.

Further, we assessed how γ-H2AX and 53BP1 were associated via their expression status, as analyzed by both IHC and IF, and we found strong positive linear associations between the H-scores of γ-H2AX and 53BP1 by IHC (Figure 4A, *p* < 0.001, r = 0.700), and between expression percentages of tumor cells exhibiting foci of γ-H2AX and 53BP1 by IF (Figure 4B, *p* < 0.001, r = 0.594). Next, the relationship between γ-H2AX and 53BP1 in the subcellular localization was analyzed by a dual-color IF assay in eight representative cases with higher expression percentages of tumor cells presenting foci of both γ-H2AX and 53BP1, in which the merged signals of γ-H2AX and 53BP1 labeling clearly demonstrated their co-localization in the form of yellow nuclear speckles (Figure 4C,D).

### 3.4. The Dosages of RB1, BRCA2, and CHEK2 Genes Determined by MLPA and Their Associations with Clinicopathologic Factors, γ-H2AX and 53BP1 in GISTs

**Figure 5:**
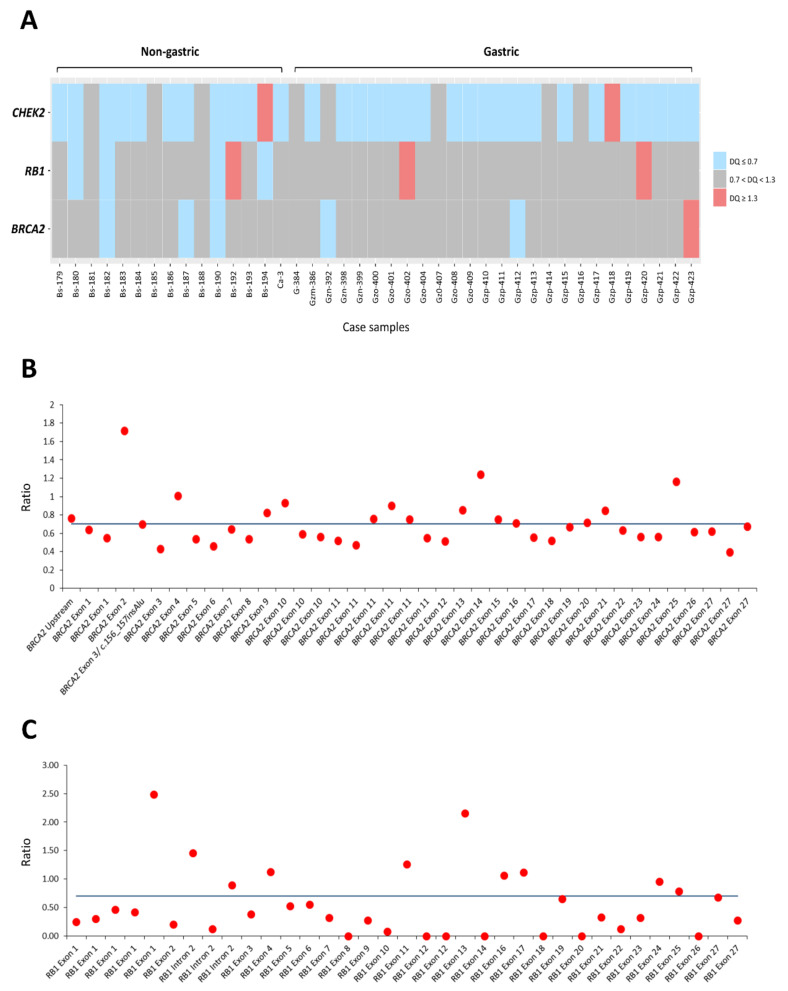
The copy number alterations of *RB1*, *BRCA2,* and *CHEK2* genes determined by MLPA assay. (**A**) The schematic illustration of MPLA assays shows frequent heterozygous deletions (HetDels) of *CHEK2* in approximately three quarters of the 41 samples tested, without significant differences between gastric and non-gastric cases. HetDels of *RB1* and *BRCA2* were much rarer than *CHEK2*-HetDel, while *RB1*-HetDel, rather than *BRCA2*-HetDel, has showed clear predominance in non-gastric GISTs. Losses of *RB1* and *BRCA2* trended toward co-deletion, but were rare events in two non-gastric cases. For each tumor tested, a gene was construed as harboring HetDel if it had a dosage quotient (DQ) of ≤0.7, and these are coded in pale blue, while normal ones are coded in grey with a DQ between >0.7 and <1.3, and duplicated ones are coded in red with a DQ ≥ 1.3. (**B**,**C**) In a representative case subjected to MPLA assays, across multiple exons, there were complex losses of *BRCA2* (**B**) and *RB1* (**C**) genes, whose DQ ratios (*Y*-axis) dropped below the blue lines.

Using DQ ≤ 0.7 as the cutoff to define deletion, 41 primary GIST samples all yielded information on the MLPA-determined copy number changes of *RB1, BRCA2,* and *CHEK2* genes (Table 2, Figure 5). As expected, given its location on 22q which commonly exhibits monosomy in GISTs, losses of *CHEK2* manifesting as HetDel prevailed in the majority of primary GISTs (75.6%, 31/41) measured by MLPA, which was more prevalent compared with the cohort profiled by targeted NGS. Regarding the MPLA-measured cohort, the *CHEK2*-deleted primary GISTs showed significantly older ages at presentation than those harboring intact *CHEK2* (*p* = 0.033, 66.03 ± 11.81 vs. 58.1 ± 9.28 years), but they showed no association with the clinicopathologic factors or expression statuses of γ-H2AX and 53BPl by either staining method, indicating their very early role in GIST inception. Compared with *CHEK2*, deletions of *RB1* and *BRCA2* were much less prevalent and were detected in only 4 (9.8%) and 5 (12.2%) of the 41 primary GISTs, respectively. Although the co-deletion of *RB1* and *BRCA2* was only seen in two cases, it showed a marginal trend toward association among all primary GISTs (*p* = 0.066), partly reflective of their chromosomal proximity. Notably, considerable variations in associations with clinicopathologic factors and γ-H2AX/53BPl were found between *RB1*-HetDel and *BRCA2*-HetDel. Similar to *CHEK2*-HetDel, *BRCA2*-HetDel was significantly associated with older age at presentation (*p* = 0.005, 76.4 ± 6.5 vs. 62.39 ± 11.22 years), while this was not the case for *RB1*-HetDel, which occurred in four cases. However, *RB1*-HetDel was significantly related to higher mitotic count (*p* = 0.005) and was exclusive to non-gastric (*p* = 0.013) and high-risk GISTs, defined by both the NIH scheme (*p* = 0.001) and NCCN guidelines (*p* = 0.003). In addition, *RB1*-HetDel was significantly associated with higher 53BPl expressions by IHC (*p* = 0.048), while *BRCA2*-HetDel only showed a marginal trend toward increased γ-H2AX expression by IF (*p* = 0.071).

### 3.5. Independent Validation of Immunohistochemical Expression, Clinical and Molecular Correlates, and Prognostic Relevance of γ-H2AX and 53BP1 in TMA-Based GISTs

In the TMA cohort, the immunostaining results of both γ-H2AX and 53 BP1 were informative for 285 GISTs (Table 3), including 188 gastric and 97 non-gastric cases, which were classified by the NIH scheme as high risk, intermediate risk, and low/very low risk in 94, 109, and 82 cases, respectively, and by the NCCN guidelines as high, moderate, low, none/very low risk, and insufficient evidence-based data for risk-stratification in 77, 64, 88, 55, and 1 case, respectively. Although higher expressers of γ-H2AX and 53BP1 were unrelated to tumor locations, both were significantly associated with the presence of epithelioid histology (γ-H2AX: *p* = 0.012; 53BP1: *p* < 0.001), higher mitotic rates (γ-H2AX: *p* = 0.021; 53BP1: *p* < 0.001), and higher risk levels by both the NIH risk scheme (γ-H2AX: *p* = 0.002; 53BP1: *p* < 0.001) and the NCCN guidelines (both *p* < 0.001). These findings are generally in keeping with those for whole sections assessed by IHC. Apart from being stronger than γ-H2AX in its associations with the above clinicopathologic variables, 53BP1, when present at high expression levels, was also significantly more commonly related to larger tumor sizes (*p* = 0.001) and 5′ deletion of the *KIT* exon 11 (*p* = 0.015).

**Figure 6:**
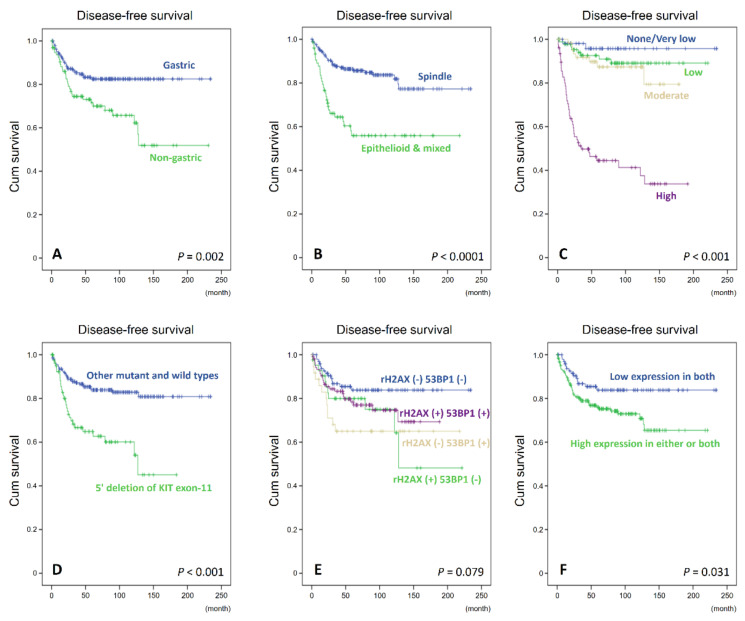
Kaplan–Meier analyses of univariate DFS in primary GISTs. The survival curves of 285 informative cases were plotted based on tumor locations (**A**), cytomorphologic features (**B**), risk levels defined by NCCN guidelines (**C**), 5′ deletion of the *KIT* exon 11 vs. other *KIT/PDGFRA* mutations (**D**), and comparisons among cases showing high expression of γ-H2AX and 53BP1 in neither, either, or both markers (**E**), and between cases showing γ-H2AX-low/53BP1-low and the cases that remain (**F**).

At the univariate level (Table 4), shorter DFS was significantly related to non-gastric locations (*p* = 0.002, Figure 6A) and strongly (all *p* < 0.001) associated with the presence of epithelioid histology (Figure 6B), increasing tumor sizes, mitotic rates, and risk levels defined by NCCN guidelines (Figure 6C), as well as with 5′ deletion of the *KIT* exon 11 (Figure 6D). Although the expression level of either γ-H2AX or 53BP1 alone did not significantly predict DFS, a trend toward improved prognostic power was observed in the combinatorial assessments of both biomarkers (*p* = 0.079, Figure 6E), and patients with GISTs featuring low expressions of both γ-H2AX and 53BP1 fared significantly better in terms of DFS than the joint group of GISTs exhibiting higher expression of either one or both markers (*p* = 0.031, Figure 6F).

Specifically, the DFS rates at 5, 10, and 15 years were all 86% in the double-low expressers, and were 77.8%, 76.2%, and 74.6% in the joint group comprising other combinations of γ-H2AX and 53BP1. Collectively, these findings indicate that the increased expression of either γ-H2AX or 53BP1, or both, might signify a perturbed genomic integrity associated with DNA damage, and represent a harbinger of progression. In multivariate analysis, a high NCCN risk level (*p* < 0.001), 5′ deletion of the *KIT* exon 11 (*p* = 0.005), and the presence of epithelioid histology (*p* = 0.033) remained clear prognostic indicators of shorter DFS, while other univariately significant factors lost their independence, including the combination of γ-H2AX and 53BP1.

## 4. Discussion

In the contemporary care of GIST patients, there is still room for improvement in the search for robust biomarkers that can outperform the currently applied risk schemes, and can overcome the inevitable resistance to kinase inhibitors [1,3,24,28]. For instance, the resistance to frontline imatinib in GISTs usually emerges following initial responses, most commonly due to secondary *KIT* mutations [28]. In addition, the primary resistance to imatinib occurs in GISTs that harbor either imatinib-refractory *KIT/PDGFRA* mutations (e.g., PDGFRA D842V) or rare alternative driver aberrations in the presence of wild-type *KIT/PDGFRA* [4,7,8,28,29]. In this study, we employed three independent cohorts of GISTs wherein the aberrations of DDR genes, focusing on *RB1*, *BRCA2,* and *CHEK2*, were correlated with expression alterations of γ-H2AX and 53BP1, in order to better understand the mutual relationships, clinicopathologic relevance, prognostic utility, and potential therapeutic implications of these biomarkers. This approach is based on the understanding that constitutive endogenous γ-H2AX and 53BP1 foci are scarce, if present at all, in normal human cells and tissues, while neoplastic cells may exhibit varying degrees of constitutive phosphorylated H2AX and 53BP1 in the absence of exogenously induced DSBs [16,17,18,30].

Genomic instability is a central hallmark of malignant transformation and is tightly linked to various genetic [10,11,12,18,31] and epigenetic [32,33] aberrations of the DDR pathways that serve as an anti-neoplastic barrier in early stages of tumorigenesis. Bi-allelic mutations of DDR-associated genes, such as *ATM*, *BRCA2*, *PALB2*, *RB1*, and *TP53*, are not only drivers of tumor evolution in cancers, but are also significantly associated with the responses to various treatment modalities, including PARP inhibitors and immunotherapies [31]. Recently, the coordinated mono-allelic loss of *BRCA2*/*RB1* on 13q was shown to evoke aggressive phenotypes in prostatic adenocarcinomas vulnerable to PARP inhibitor-based therapy [21]. Our targeted NGS profiling detected various SNVs of multiple DDR genes involving DNA damage-sensing, HRR, and Fanconi anemia pathways, which were missense in 31.2% and splicing in 25% of the 16 GISTs analyzed, while they were largely indefinitely pathogenic or insufficiently recurrent with only sporadic occurrence in no more than two cases. Only in a single GIST was *RB1* p.E492K found to co-occur with mono-allelic HetDel, which should lead to full inactivation in two hits. In our NGS-profiled GISTs, HetDels represented the major mode of recurrent DDR gene aberrations, in which the simultaneous involvement of multiple genes and DDR pathways was commonly detected even in a single case. Overall, HetDels most frequently occurred in the genes responsible for DNA damage sensing, including *CHEK2* in 37.5%, *BLM* in 31.3%, *ATM* in 25%, *CHEK1* in 18.8%, and *ATR* in 6.3%. However, *RB1* (25%), as well as genes involved in the HRR (e.g., *RAD51* in 18.8%) and Fanconi anemia (e.g., *BRCA2* in 12.5%) pathways, were also recurrently hit by HetDels. These findings are largely in keeping with a recent whole-exome sequencing study on GISTs, which demonstrated a potential relationship between CNVs and DDR [34]. Therefore, the CNV-driven deregulation of DDR gene expression has been proposed as a mechanism of defective DNA repair, while sporadic pathogenic missense mutations of DDR genes, e.g., *BRCA1, BRCA2,* and *POLE,* were also identified in that study. Despite their higher rate of copy number losses, no *CHEK2* SNVs or Indels, and a considerably low rate of *RB1* pathogenic SNVs (mean: 4.5%; range: 2.4~13.6% in 223 cases), were identified after a thorough review of previous NGS studies on GISTs [35,36,37,38]. Importantly, previously documented GISTs harboring *RB1* pathogenic SNVs tended to be enriched in imatinib-resistant, high-risk or metastatic cases.

Of the genes hit by HetDel in the current study, *CHEK2* codes for a serine/threonine kinase, which is rapidly phosphorylated by ATM in response to DNA damage by recruiting BRCA1, and in response to replication blocks by stabilizing p53 to halt cell cycle progression [12,13,31,39]. In vivo, the crucial role of CHEK2 in mediating the DDR signal was exemplified in a knockout mice model, exhibiting G1/S checkpoint defect and prominent radioresistance [40]. Similarly to the role of ATM in cellular senescence, *CHEK2* is a multiorgan tumor susceptibility gene involved in the induction of replication- and oncogene-triggered senescence to block hyperproliferation and subsequent tumorigenesis [41,42]. Clinically, *CHEK2* deletion has previously been shown to be associated with breast cancers via a combined assay using NGS and MPLA [39]. Regarding DDR genes, *CHEK2*-HetDel was the most prevalent aberration in GISTs, as detected by both the NGS and MLPA assays, although the frequency of this aberration was apparently higher in the MPLA-measured cohort. This discrepancy might be attributable to the differences in the sample sizes and compositions between cohorts, and/or to the MLPA probe panel applied, which covered fewer exons of *CHEK2,* although the detected DQ value of *CHEK2* in the MPLA assay largely corresponded to heterozygous losses. Despite only the mono-allelic deletion of *CHEK2* being detected, without concomitant SNVs, the possibility of the functional abrogation of *CHEK2* in GISTs cannot be excluded, since *CHEK2* may participate in tumorigenesis via haploinsufficiency, and behave differently from the classical tumor suppressor gene defined by Kundson [42,43]. In addition, prior studies have reported epigenetic silencing (e.g., *CHEK2* promoter methylation) as a mechanism of downregulating CHEK2 expression in other cancer types [44,45,46].

Compared with *CHEK2*, both *RB1* and *BRCA2* were relatively infrequently hit by HetDels, with this occurring in 25% and 12.5% of the NGS-profiled cases and in 9.8% and 12.2% of the MPLA-measured cases, respectively. Notably, mono-allelic *RB1/BRCA2* co-deletion was detected in 12.5% of GISTs by targeted NGS. Despite the marginal trend toward significance, the frequency of *RB1/BRCA2* co-deletion was even lower (~5%, 2/41) in GISTs analyzed by MLPA, indicating independent alterations of either gene in a subset of primary GISTs. In the MPLA assay, the *BRCA2*-HetDel, similarly to the *CHEK2*-HetDel, showed a predilection for older ages at presentation, without significant relations to other clinicopathologic variables. Nevertheless, similarly to *CHEK2*-HetDel, the age preferences of *BRCA2*-HetDel cannot be simply explained by the accumulation of mutations because of aging, as *CHEK2* has intricate functional links to the modulation of the expression and/or activity of both BRCA2 and RB1 in DNA repair, cell cycle arrest, and senescence [41,42,47]. In contrast, the *RB1*-HetDel in GISTs exhibited significant associations with multiple parameters, including non-gastric location, increased mitosis, higher risk levels and higher 53BP1 H-score. These findings imply that *RB1*-HetDel may differ from others involving the closely located *BRCA2* and/or *CHEK2,* in their impact on GIST evolution. *RB1* is a well-established tumor suppressor, which not only governs progression across the G1 checkpoint, but also instigates DNA damage repair and GIN as novel functions to restrain tumor progression [48,49]. In cooperation with E2F1, RB may moonlight as a DNA repair factor by co-recruiting chromatin-modifying enzymes so as to enhance the accessibility to chromatins, hence protecting genomic stability [48,49]. From a pan-cancer perspective, although the bi-allelic deletion of *RB1* is relatively uncommon, some aspects of non-canonical pathways, e.g., DNA damage repair, appear to be sensitive to single-copy *RB1* loss [21]. Moreover, *Rb1* haploinsufficiency arising with the loss of a single copy of the *Rb1* gene was found to promote telomeric attrition and genomic instability in cells of osteoblastic lineage [50].

Although IF is traditionally considered the gold standard for determining expression alterations in γ-H2AX and 53BP1 [17,18], our study, using archival GIST samples, clearly demonstrated robust and positive correlations between the expression percentages given by IF and H-scores given by IHC for both γ-H2AX and 53BP1. Furthermore, the strong association between γ-H2AX and 53BP1, obtained by both IF and IHC staining, was mirrored by the nuclear co-localization of γ-H2AX and 53BP1 in a dual-color IF assay. Given the practicability of IHC in a diagnostic work-up, our findings indicate the utility of IHC assays of γ-H2AX and 53BP1 as potential surrogates for more laborious IF assays. Indeed, the H-scores of both γ-H2AX and 53BP1 in the joint group of none/very low-/low-risk GISTs were significantly higher than those of adjacent normal tissues, but were noticeably lower than those of combined moderate-risk and high-risk cases. Similarly, variations in the IF expression levels of γ-H2AX and 53BP1 were also highly significant when compared among normal tissue and two combined groups comprising GISTs at different risk levels. In every case, high expressers of γ-H2AX and those of 53BP1 were significantly associated with increased proportions of cases at higher risk levels, regardless of whether IHC or IF staining was used. Besides this, the higher H-score of γ-H2AX determined by IHC was significantly related to intestinal, mitotically active, and larger GISTs, while the high γ-H2AX expressers assessed by IF were only significantly associated with higher mitotic activity. These differences seemingly imply the better performance of γ-H2AX IHC compared to γ-H2AX IF, while this was not the case for 53BP1. In the TMA-based large cohort, the higher H-scores of 53BP1 and γ-H2AX given by IHC were shown to be significantly associated with several adverse clinicopathologic factors, such as epithelioid histology and higher risk levels. Intriguingly, only high expressers of 53BP1 were predominant in GISTs >5 cm and those harboring 5′ deletions of the *KIT* exon 11. Given the above pros and cons and the comparisons among both markers at various expression levels in terms of predicting DFS, a combined assessment of both 53BP1 and γ-H2AX by IHC should better reflect the intrinsic biology and prognostic relevance of GISTs. Specifically, GISTs featuring low expression levels of both makers were related to a significantly more favorable DFS than those exhibiting increased expressions of either 53BP1 or γ-H2AX, or both.

**Figure 7:**
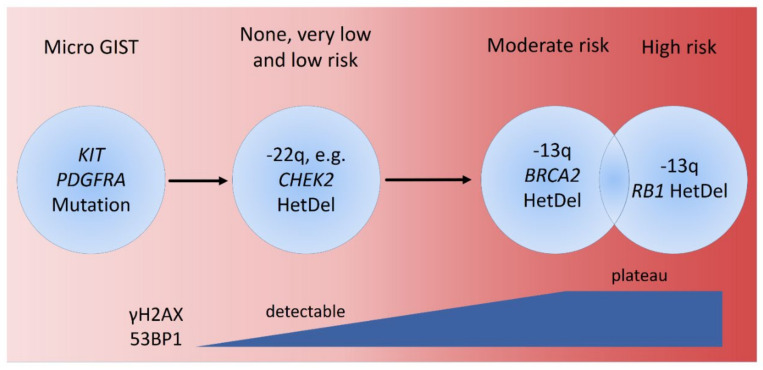
Schematic model diagram illustrates the timing of accumulation of γ-H2AX and 53BP1 in relation to heterozygous deletions of DNA damage repair genes during tumor evolution toward malignancy.

In conclusion, the SNVs of genes in the DDR pathways are often insufficiently recurrent and usually indefinitely pathogenetic, as profiled by NGS. In contrast, HetDels represent the most common form of aberrations in DDR genes, and the highly frequent *CHEK2*-HetDel may play an early inceptive role in GISTs (Figure 7). However, *RB1* and *BRCA2* can be separately deleted or concomitantly lost in a heterozygous manner in a minor subset of high-risk cases, often accompanied by deregulated 53BP1 and/or γ-H2AX. γ-H2AX and 53BP1 foci begin accumulating in the no/very low-/low-risk cases as early events in the evolution of GIST, and reach similarly high expression levels in moderate-risk and high-risk cases. As a potential surrogate of the more laborious IF examination, the immunohistochemical evaluation of γ-H2AX and 53BP1 is applicable to GIST samples in which higher risk levels, amongst other adverse factors, are robustly associated with increased expressions of both markers. In addition, the utility of a combined assessment of both 53BP1 and γ-H2AX by IHC is justified. These features are in accordance with the role of 53BP1, in cooperation with γ-H2AX, in signifying DNA repair and GIN, while functional validation is warranted to corroborate the biological, and perhaps therapeutic, relevance of recurrently aberrated DDR genes in GISTs.

## Figures and Tables

**Table 1:** Associations of various clinicopathologic parameters with rH2AX and 53BP1 expression levels by IHC and IF.

Parameters	rH2AX H-Score		*p*-Value	rH2AX IF		*p*-Value	53BP1 H-Score		*p*-Value	53BP1 IF		*p*-Value
	Low	High		Low	High		Low	High		Low	High	
**Sex**			0.121			0.825			0.237			0.121
Male	21	28		24	25		26	23		28	21	
Female	21	14		18	17		14	21		14	21	
**Age (years) ^&^**	60.5 ± 12.821	64.19 ± 12.451	0.243	60.5 ± 11.171	64.19 ± 13.952	0.088	60.7 ± 13.885	63.84 ± 11.471	0.373	62.48 ± 12.547	62.21 ± 12.998	0.9
**Location**			0.005 *			0.637			0.514			0.3445
Gastric	35	23		30	28		29	29		27	31	
Non-gastric	7	19		12	14		11	15		15	11	
**Histologic Type**			0.266			0.266			0.145			0.578
Spindle	36	32		36	32		35	33		35	33	
Epithelioid and mixed	6	10		6	10		5	11		7	9	
**Tumor size (cm)**	3.785 ± 1.877	6.227 ± 4.803	0.01 *	4.212 ± 2.538	5.8 ± 4.681	0.128	4.128 ± 2.25	5.843 ± 4.757	0.091	4.622 ± 3.577	5.390 ± 4.068	0.244
<5 cm	33	23	0.018 *	31	25	0.154	30	26	0.203	30	26	0.342
>5 cm	8	18		10	16		10	16		11	15	
**Mitotic count (50HPFs) ^&^**	1.74 ± 1.149	8.46 ± 9.998	<0.001 *	2.91 ± 6.818	7.26 ± 8.201	<0.001 *	3.08 ± 6.893	6.91 ± 8.214	0.016 *	2.95 ± 6.811	7.22 ± 8.233	0.001 *
**NIH Risk**			<0.001 *			0.001 *			0.001 *			0.007 *
Low/very low	34	14		32	16		30	18		31	17	
Intermediate	8	9		7	10		8	9		6	11	
High	0	19		3	16		2	17		5	14	
**NCCN guideline**			<0.001 *			<0.001 *			<0.001 *			0.002 *
None/very low	29	11		25	15		25	15		24	16	
Low	12	4		13	3		11	5		12	4	
Moderate	1	10		2	9		2	9		2	9	
High	0	17		2	15		2	15		4	13	

*, Statistically significant; ^&^, Wilcoxon rank-sum test; IF, immunofluorescence expression; NIH, National Institute of Health; NCCN, National Comprehensive Cancer Network; HPFs, high power fields.

**Table 2:** Associations of *RB1, BRCA2, and CHEK2* gene dosages with various clinicopathologic parameters.

Parameters	*RB1* Dosage		*p*-Value	*BRCA2* Dosage		*p*-Value	*CHEK2* Dosage		*p*-Value
	DQ > 0.7	DQ ≤ 0.7		DQ > 0.7	DQ ≤ 0.7		DQ > 0.7	DQ ≤ 0.7	
**Sex**			0.107			0.564			0.201
Male	26	1		24	3		5	22	
Female	11	3		12	2		5	9	
**Age (years) ^&^**	64.08 ± 11.15	64.25 ± 17.86	1	62.39 ± 11.22	76.4 ± 6.5	0.005 *	58.1 ± 9.28	66.03 ± 11.81	0.033 *
**Location**			0.013 *			0.249			0.54
Gastric	26	0		24	2		6	20	
Non-gastric	11	4		12	3		4	11	
**Histologic Type**			0.288			0.11			0.546
Spindle	28	2		28	2		7	23	
Epithelioid and mixed	9	2		8	3		3	8	
**Tumor size (cm)**			0.283			0.308			0.475
<5 cm	23	1		22	2		6	18	
>5 cm	13	3		13	3		3	13	
**Mitotic count (50HPFs) ^&^**	4.14 ± 5.75	15.5 ± 12.58	0.005 *	5.11 ± 7.64	6.4 ± 5.18	0.449	7.4 ± 10.8	4.57 ± 5.83	0.89
**NIH Risk**			0.001 *			0.086			0.405
Low/very low	17	0		15	2		3	14	
Intermediate	14	0		14	0		3	11	
High	6	4		7	3		4	6	
**NCCN guideline**			0.003 *			0.23			0.397
None/very low	14	0		13	1		4	10	
Low	10	0		9	1		1	9	
Moderate	7	0		7	0		1	6	
High	6	4		7	3		4	6	
**rH2AX H-score**			0.143			0.341			0.612
Low	16	0		15	1		4	12	
High	21	4		21	4		6	19	
**rH2AX IF**			0.143			0.071			0.325
Low	16	0		16	0		5	11	
High	21	4		20	5		5	20	
**53BP1 H-score**			0.048 *			0.156			0.607
Low	21	0		20	1		5	16	
High	16	4		16	4		5	15	
**53BP1 IF**			0.118			0.258			0.535
Low	18	0		17	1		4	14	
High	19	4		19	4		6	17	

* Statistically significant; ^&^ Wilcoxon rank-sum test; IF, immunofluorescence expression; NIH, National Institute of Health; NCCN, National Comprehensive Cancer Network; HPFs, high power fields.

**Table 3:** Immunohistochemistry of rH2AX and 53BP1 in the tissue microarray-based cohort: associations of expression levels with clinicopathologic and mutation variables.

Parameters	rH2AX H-Score		*p*-Value	53BP1 H-Score		*p*-Value
	Low	High		Low	High	
**Sex**			0.889			0.372
Male	65	69		63	71	
Female	72	79		79	72	
**Age (years) ^&^**	60.57 ± 12.36	59.12 ± 12.01	0.395	58.82 ± 13.35	60.8 ± 10.94	0.158
**Location**			0.097			0.359
Gastric	97	91		90	98	
Non-gastric	40	57		52	45	
**Histologic Type**			0.012 *			<0.001 *
Spindle	108	98		118	88	
Epithelioid and mixed	27	49		23	53	
**Tumor size (cm)**			0.081			0.001 *
≤5 cm	63	53		72	44	
>5 cm	74	95		70	99	
**Mitotic count (50HPFs) ^&^**	8.72 ± 23.68	9.95 ± 21.95	0.021 *	4.79 ± 16.08	13.89 ± 27.17	<0.001 *
**NIH Risk**			0.002 *			<0.001 *
Low/very low	52	30		63	19	
Intermediate	50	59		51	58	
High	35	59		28	66	
**NCCN guideline ^#^**						
None/very low	38	17	<0.001 *	41	14	<0.001 *
Low	51	37		60	28	
Moderate	19	45		17	47	
High	28	49		23	54	
**Genotypes**			0.449			0.015 *
Other mutant and wild types	100	102		110	92	
5′ deletion of KIT exon-11	37	46		32	51	

*, Statistically significant; ^&^, Wilcoxon rank-sum test; NIH, National Institute of Health; NCCN, National Comprehensive Cancer Network; HPFs, high power fields. ^#^, One of the 285 GISTs lacked sufficient evidence-based data for risk stratification according to the NCCN guideline.

**Table 4:** Univariate and multivariate analyses for disease-free survival in 285 GISTs of the tissue microarray-based cohort.

Parameters	Univariate Analysis			Multivariate Analysis		
	No. Case	No. Event	*p*-Value	HR	95% CI	*p*-Value
**Sex**			0.563			
Male	134	30				
Female	151	31				
**Age (years)**			0.1			
<70	214	41				
≥70	71	20				
**Location**			0.002 *			0.237
Gastric	188	30		1	-	
Non-gastric	97	31		1.365	0.815–2.289	
**Histologic Type**			<0.0001 *			0.033 *
Spindle	206	32		1	-	
Epithelioid and mixed	77	29		1.8	1.048–3.093	
**Tumor size (cm)**			<0.001 *			
≤5 cm	116	10				
>5; ≤10 cm	119	25				
≥10 cm	50	26				
**Mitotic count (50HPFs)**			<0.001 *			
0–5	197	24				
6–10	43	9				
>10	45	28				
**NCCN guideline ^#^**			<0.001 *			<0.001 *
None/very low	55	2		1	-	
Low	88	8		2.05	0.431–9.743	
Moderate	64	8		2.518	0.514–12.339	
High	77	43		14.612	3.355–63.641	
**Genotypes**			<0.001 *			0.005 *
Other mutant and wild types	202	32		1	-	
5′ deletion of KIT exon-11	83	29		2.12	1.23–3.568	
**rH2AX expression**			0.403			
Low expression	137	26				
High expression	148	35				
**53BP1 expression**			0.125			
Low expression	142	25				
High expression	143	36				
**Combinations of rH2AX and 53BP1**			0.031 *			0.537
Low expression in both	100	14		1	-	
High expression in either or both	185	47		0.815	0.426–1.559	

Tumor size and mitotic activity were not introduced into the multivariate analysis, since these two parameters were component factors of the NCCN risk scheme; *, Statistically significant; HR, hazard ratio; CI, confidence intervals; 53BP1, P53-binding protein 1; GISTs, gastrointestinal stromal tumors; NCCN, National Comprehensive Cancer Network; HPFs, high power fields. ^#^, 1 of the 285 GISTs lacked sufficient evidence-based data for risk stratification according to the NCCN guideline.

## Data Availability

The data presented in this study are available on request from the corresponding author (H.-Y.H.).

## Supplementary Materials

The following supporting information can be downloaded at: https://www.mdpi.com/article/10.3390/cancers14071787/s1, Figure S1: Mutational landscape of GISTs in nucleotide excision repair (NER) and non-homologous end-joining (NHEJ) pathways: In the targeted NGS panel, two genes in the NER pathway (ERCC3: p.K11E (*n* = 2) and p.S764L (*n* = 1); *ERCC5*: p.R71H (*n* = 1)) and one gene in the NHJE pathway (*PRKDC*: p.M3845I, p.P2456A, and p.G1030V in 1 each), all with missense mutations, are aberrated in 4 and 3 cases, respectively. Columns represent individual tumors and rows represent individual aberrated genes. DNA damage repair genes are collated based on the prevalence of SNVs/indels, following the primary GIST-driving mutations (*KIT, PDGFRA, NF1, SDHC*) and the baseline clinicopathologic characteristics, including tumor locations, NCCN risk levels, and status of tumor specimens. Asterisks indicate biopsy samples not amenable to precise risk-stratification by histology.
