# Supplementary material for: Characterization of Aberrations in DNA Damage Repair Pathways in Gastrointestinal Stromal Tumors: The Clinicopathologic Relevance of γH2AX and 53BP1 in Correlation with Heterozygous Deletions of CHEK2, BRCA2, and RB1
Source: Cancers (Basel). 2022 Mar 31;14(7):1787. doi: 10.3390/cancers14071787 (PMC8997382; doi:10.3390/cancers14071787)
Supplement: Supplementary file 1 [file cancers-14-01787-s001.zip › cancers-1633621-supplementary.pdf]

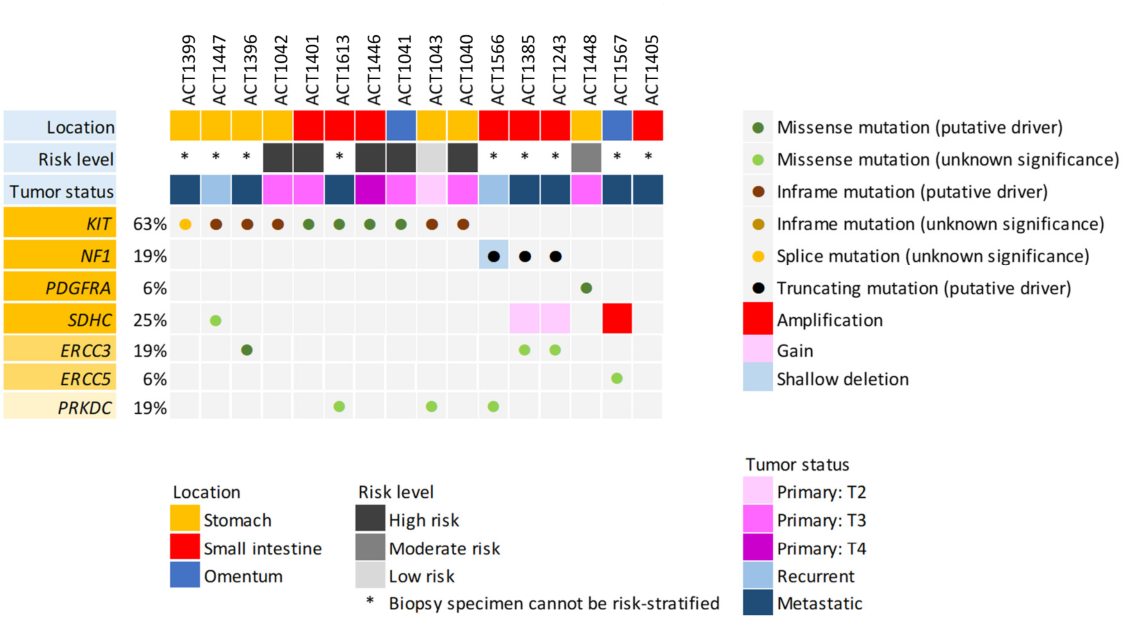

Figure S1. Mutational landscape of GISTs in nucleotide excision repair (NER) and non-homologous end-joining (NHEJ) pathways: In the targeted NGS panel, two genes in the NER pathway (ERCC3: p.K11E (*n* = 2) and p.S764L (*n* = 1); *ERCC5*: p.R71H (*n* = 1)) and one gene in the NHJE pathway (*PRKDC*: p.M3845I, p.P2456A, and p.G1030V in 1 each), all with missense mutations, are aberrated in 4 and 3 cases, respectively. Columns represent individual tumors and rows represent individual aberrated genes. DNA damage repair genes are collated based on the prevalence of SNVs/indels, following the primary GIST-driving mutations (*KIT, PDGFRA, NF1, SDHC*) and the baseline clinicopathologic characteristics, including tumor locations, NCCN risk levels, and status of tumor specimens. Asterisks indicate biopsy samples not amenable to precise risk-stratification by histology.
